# Cementless modular total hip arthroplasty with subtrochanteric transverse shortening osteotomy for high hip dislocations

**DOI:** 10.1186/s13018-022-03025-4

**Published:** 2022-03-04

**Authors:** Erhan Sukur, Ahmet Senel, Ugur Ozdemir, Yunus Emre Akman, İbrahim Azboy, Yusuf Ozturkmen

**Affiliations:** 1 Departments of Orthopaedics and Traumotology, Sakarya University Training and Research Hospital, 54050 Sakarya, Turkey; 2 Departments of Orthopaedics and Traumotology, Istanbul Training and Research Hospital, Istanbul, Turkey; 3 Departments of Orthopaedics and Traumotology, Ortopedkliniken Mälarsjukhuset, Eskilstuna, Sweden; 4 Departments of Orthopaedics and Traumotology, Medipol University Hospital, Istanbul, Turkey

**Keywords:** High hip dislocation, Modular, Transverse osteotomy

## Abstract

**Background:**

Performing a total hip arthroplasty for a high hip dislocation is technically demanding and presents several challenges, with appropriate evaluation of the bone morphology of the hip and proper implant selection being critical for successful outcomes.

**Objective:**

The purpose of our study was to evaluate the clinical and radiographic outcomes of cementless modular total hip arthroplasty for the treatment of high hip dislocations with sub-trochanteric transverse shortening osteotomy.

**Methods:**

Sixty-eight hips with a high hip dislocation, were treated using a sub-trochanteric transverse shortening osteotomy and cementless modular total hip arthroplasty, retrospectively reviewed. Hip function was assessed using the Harris hip score, with hip abduction strength evaluated using the Trendelenburg test. Radiographic assessment included the measurement of leg length discrepancy, identification of implant loosening, localization of the hip center, and union at the osteotomy site.

**Results:**

The mean follow-up period was 12.9 (range 5.2–16.8) years. The mean Harris hip score improved from a pre-operative score of 48.6 ± 3.6 to 82.4 ± 4.2 (*p* < 0.05). The hip was within the true acetabulum in all patients, postoperatively. Osteotomy union was achieved in 67 of the 68 hips (98.5%) in a mean latency of 12.5 ± 0.6 weeks. The mean length of femoral shortening was 66.5 ± 4.5 mm, with a mean pull-down length of the proximal part of 35.5 ± 3.5 mm.

**Conclusion:**

For the treatment of high hip dislocations with satisfactory clinical outcomes, the modular stem offers an adjustable anteversion angle to restore sufficient rotational stability and the transverse osteotomy provides sufficient compression pressure across the osteotomy site to facilitate union.

*Trial registration* It was a retrospective study and approved by Istanbul Research and Training Hospital institutional Ethics Review Board (772-05/02/2016).

## Background

Performing total hip arthroplasty (THA) for high hip dislocation is technically demanding and presents several challenges to surgeons. Indeed, appropriate evaluation of the hip bone structure and proper implant selection are critical to successful outcomes [1–3]. The difficulties in identifying and preparing the acetabulum and achieving stable fixation of the acetabular component are well described in the literature [1, 4–8]. When considering the femur, the most notable morphological variations are excessive femoral anteversion and a high valgus neck-shaft angle that reduces acetabular coverage of the femoral head [1]. Moreover, dysplastic femurs have a small diaphyseal diameter that requires careful stem selection [9].

A key component of acetabular reconstruction is the restoration of the anatomical hip center, with reduction of the femoral head into the true acetabulum being important in achieving better hip biomechanics and bone support for implantation, as well as longer THA survival [3, 4, 10]. However, achieving and maintaining reduction of the femoral head into the true acetabulum is difficult in patients with high hip dislocations due to severe soft tissue contracture, leg length discrepancy, impairment in abductor muscle function, and abnormal bone morphology [10]. Sub-trochanteric osteotomy and femoral shortening have been performed using a pull-down technique to address these complications [3, 11–13]. In these cases, achieving rotational stability and compression at the osteotomy site is essential in preventing non-union [14]. However, obtaining a straight femoral canal and selecting the appropriate fit and fill implant is difficult using mono-block femoral implants [4]. Fitting of the femoral implant is further complicated by the sub-trochanteric osteotomy itself, which produces incongruence between the diameters of the metaphyseal and diaphyseal canals, increasing the risk for non-union [4, 15–18].

A modular femoral stem can improve fitting in a straight and narrow femoral canal, stabilizing both the metaphyseal and diaphyseal components to provide sufficient torsional stability across the osteotomy site [11]. Additionally, the different pairing of sleeve and handle of the femoral components with a modular stem is beneficial to correct excessive anteversion [11]. Therefore, we aimed to evaluate the clinical and radiographic outcomes of cementless modular THAs to treat high hip dislocations with sub-trochanteric transverse shortening osteotomy.

## Materials and Methods

This retrospective study was approved by Istanbul Research and Training Hospital institutional Ethics Review Board (2016-772). Between October 2001 and February 2012, 56 patients (68 hips) with Crowe type IV—Hartofilakidis type III high hip dislocations were treated using a sub-trochanteric, transverse shortening osteotomy, and cementless modular THA [19, 20]. All patients were female, with a mean age of 48.2 (range 36–64) years. Forty-four patients (78.6%) presented with unilateral hip dysplasia, with the remaining 12 (21.4%) presenting with bilateral hip dysplasia. There was a 3-month interval between the two surgeries. Relevant demographic and pre-operative clinical features of the study group are summarized in Table 1. The indications for THA included severe hip pain and functional impairment in daily activities, as well as abnormal gait due to leg length discrepancy and abductor weakness.

**Table 1:** Demographic data of patients

Number of patients/hips	56/68
Bilateral/unilateral hip	12 (21.4%)/44 (78.6%)
Age (years)	48.2 (range 36–64)
Gender; female/male	56/0
Mean follow up (years)	12.9 (range 5.2–16.8)

Routine follow-up examinations were performed in the first, fourth, and twelfth months postoperatively. The final status of the patients was obtained from the latest available outpatient records. Pre-operative clinical and radiographic assessments were performed during the latest follow-up examinations. Hip function and hip abductor strength were assessed using the Harris hip score (HHS) and the Trendelenburg test, respectively [21]. Leg length was measured as the distance between the anterior superior iliac spine and the medial malleolus. Complications were noted at each follow-up visit.

The radiographic assessment included anteroposterior and lateral hip views to measure leg length discrepancy after surgery, identification of implant loosening and migration, localization of the anatomical hip center, and determination of bony union at the osteotomy site. The change in leg length from pre- to post-operation was calculated using the pulled down distance (vertical distance from the tip of the greater trochanter to the teardrop line) and the length of femoral shortening [10]. The location of the anatomical hip center was defined as the vertical distance between the ischial tuberosity and the center of the femoral head. Femoral component stability was classified according to the criteria of Engh et al. [22] as follows: fixation by bony ingrowth, stable fibrous ingrowth and unstable fibrous ingrowth. Serial radiographs were assessed by a single surgeon to evaluate union (bridging of the bone trabeculae) at the osteotomy site.

**Fig. 1:**
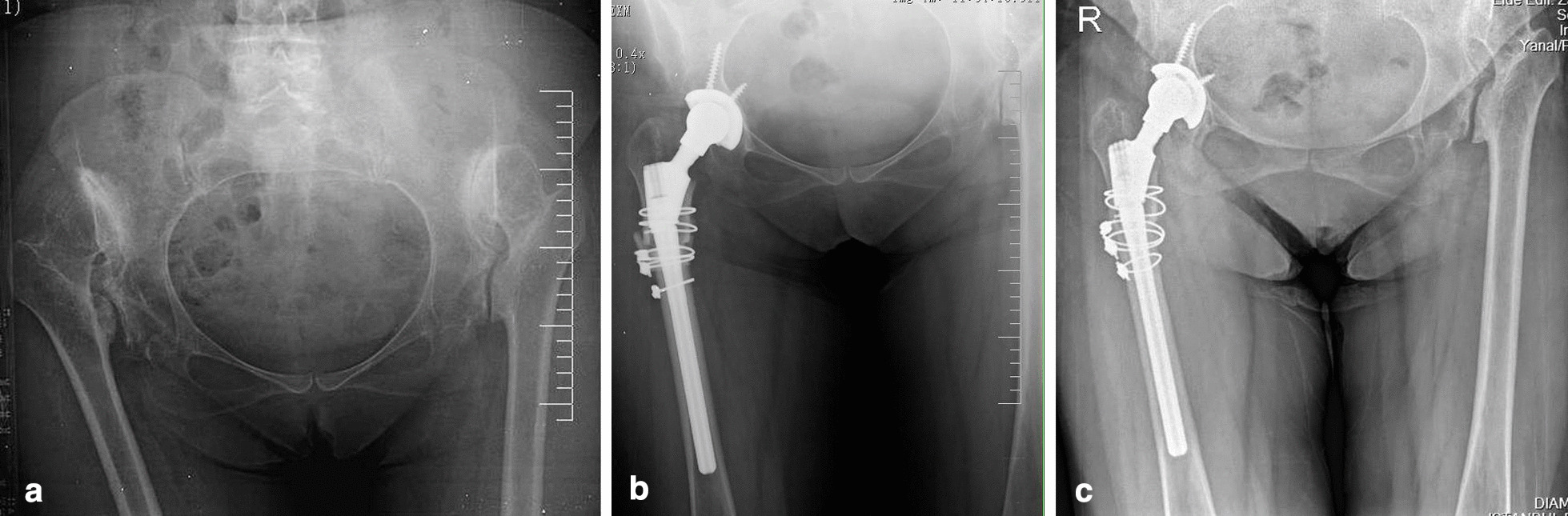
Radiographs of a 54 years old female with high hip dislocation. **a** Preoperative anteroposterior view. **b** Early postoperative radiographic image. Hip was reconstructed at the level of the anatomic hip center by cementless modular (Helios, Valencia, Biomet, Spain) total hip arthroplasty combined with transverse osteotomy and the osteotomy site was wrapped with the osteotomy fragments, stabilized using cerclage wires. Reconstruction of the acetabulum with screws was performed. **c** Postoperative radiographic image at 16-month follow-up, bone union was detected at the osteotomy site and no radiolucent lines around the femoral stem were found

**Fig. 2:**
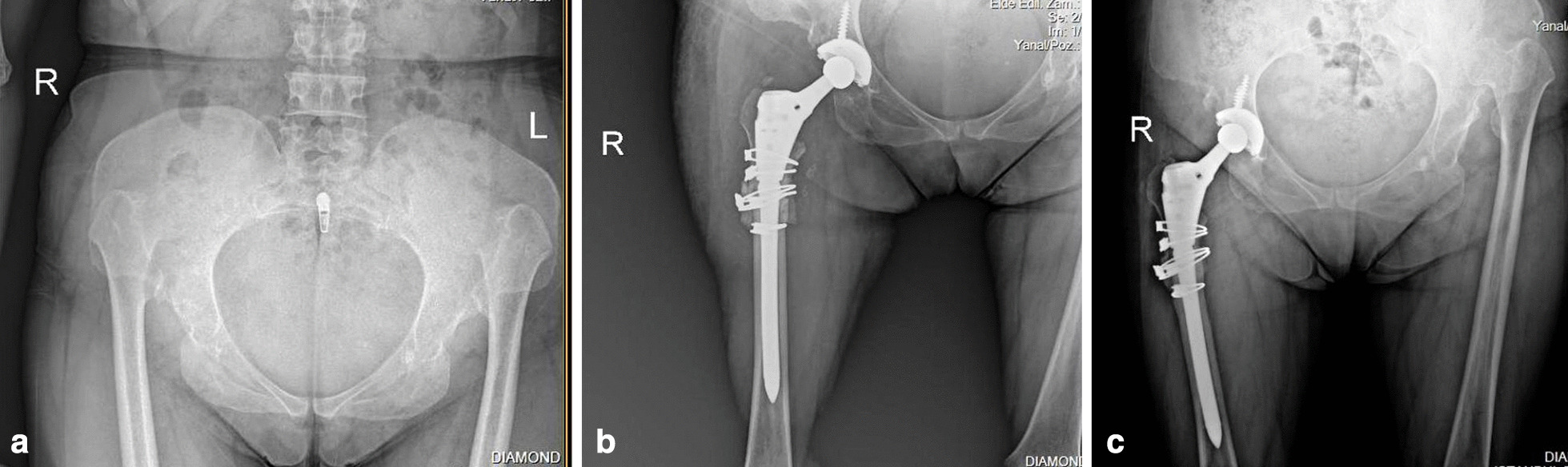
Radiographs of a 48 years old female with high hip dislocation. **a** Preoperative anteroposterior view. **b** Early postoperative radiographic image. Hip was reconstructed at the level of the anatomic hip center by cementless modular (Arcos, Zimmer, Biomet) total hip arthroplasty combined with transverse osteotomy and the osteotomy site was wrapped with the osteotomy fragments, stabilized using cerclage wires. **c** Postoperative radiographic image at 3 years follow-up, bone union was detected at the osteotomy site and no radiolucent lines around the femoral stem were found

Two femoral components were used in this study: the straight cylindrical cementless component with a proximal porous coating (Helios, Valencia, Biomet, Spain) (Fig. 1) was used in 39 patients (47 hips) between 2001 and 2011, and the modular femoral revision stem (Arcos, Zimmer, Biomet) (Fig. 2) in 17 patients (21 hips) between 2011 and 2012. A porous-coated acetabular component with a dome screw (Exceed AB Acetabular Cup, Zimmer, Biomet) was used in all patients with metal-on-polyethylene bearing surfaces.

### Surgical technique

A posterolateral approach was used with distal extension to access the proximal femoral shaft. The sciatic nerve was identified and protected. Following total capsulectomy, reaming and rasping of the femoral canal was performed before femoral osteotomy and acetabular preparation. The surface of the femoral shaft was marked with a bone chisel, from proximal to distal, to guide rotation of the femoral shaft during implantation. A transverse osteotomy was performed using a motor saw, approximately 1 cm below the inferior surface of the lesser trochanter and perpendicular to the femoral shaft as previously described by Masonis et al. [17]. The proximal fragment of the femur was retracted in a cephalo-lateral direction to facilitate soft tissue dissection and provide adequate exposure of the acetabulum. The acetabular component was positioned in the true acetabular region, as described by Sener et al. [16], with the true acetabulum identified by dissecting the joint capsule to the inferior of the acetabulum to locate the transverse acetabular ligament. The acetabular component was then implanted in the true acetabulum and fixed with a dome screw. The proximal part of the femoral component having the largest offset and best fit in the proximal fragment was selected. Once implanted, a trial hip reduction was performed to determine where to cut the distal part of the femur overlapping with the proximal part. The overlap length of bone was removed by osteotomy. The amount of femoral shortening was planned so that the lower limb would be lengthened by no more than 3 cm. The resected bone fragment was divided in the midline and opened like a book for wrapping around the osteotomy site. The canal of the distal femoral fragment was prepared using rasping until a secure fixation was obtained. After reduction with the actual stem, the rigid rotational stability of the stem was checked at both the proximal and distal sites. Efforts were made to ensure maximum bone contact at the osteotomy line. Additionally, the osteotomy site was wrapped with the osteotomy fragments and stabilized using 2 cables in most cases.

### Statistical analysis

Two-sided, paired Student’s *t*-test was used to analyze pre- and postoperative HHS and clinical and radiological measurements after confirming the normal distribution and equal variance of the data. Statistical significance was set at *p* < 0.05.

## Results

Demographic data of enrolled patients are summarized in Table 1. The mean follow-up period was 12.9 (range 5.2–16.8) years. The mean HHS improved from a pre-operative score of 48.6 ± 3.6 to 82.4 ± 4.2 at the most recent follow-up time point (*p* < 0.05; Table 2).

**Table 2:** Clinical results

	Pre-operative	Postoperative	*p-value
**Harris Hip score**			
Mean score (range)	48.6 ± 3.6 (34–66)	82.4 ± 4.2 (72–92)	< 0.05
Rating (*n*)			
Excellent	0	22	
Good	5	23	
Fair	15	5	
Poor	34	4	
**Limp (*n*)**			
Severe	36	4	
Moderate	10	6	
Slight	8	25	
None	0	19	
**Limb length discrepancy**			
Mean (range) (mm)	43 (35–60)	5 (1–15)	< 0.005
**(*n*)			
< 1 cm	0	37	
1–2 cm	0	7	
> 2 cm	44	0	
**Positive Trendelenburg sign (*n*)**	54	11	

*Statistical significance was set at *p* < 0.05, **Recorded only for patients with unilateral high hip dislocations

**Fig. 3:**
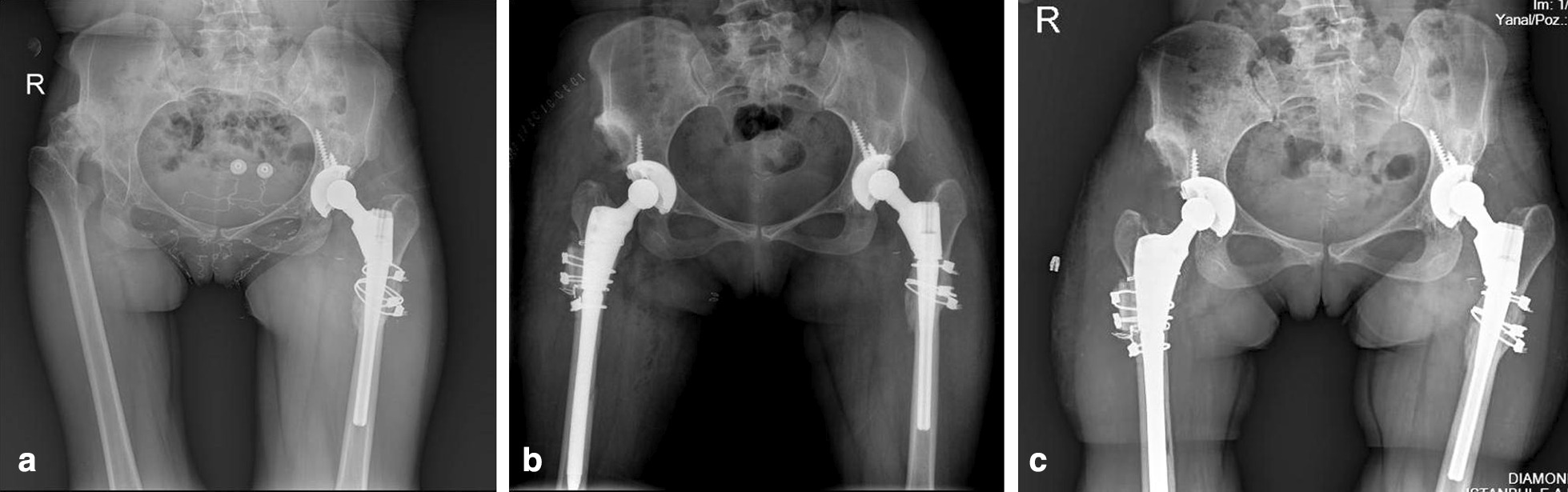
Radiographs of a 62 years old female with high hip dislocation. **a** Preoperative anteroposterior view. **b** Early postoperative radiographic image. Asetabuler componenet was reconstructed in the true acetabulum with transverse osteotomy. **c** Postoperative radiographic image at 5 years follow-up, bone union was detected at the osteotomy site

The postoperative anatomical hip center was within the true acetabulum in all patients (Fig. 3a, b, c). Osteotomy union was achieved in 67 of the 68 hips (98.5%) in a mean latency of 12.5 ± 0.6 weeks (Figs. 1, 2, 3). The mean length of femoral shortening was 66.5 ± 4.5 mm, with a mean pull-down length of the proximal part of 35.5 ± 3.5 mm. While the pre-operative number of patients with a positive Trendelenburg sign was 54, this number was reduced to 11 after surgery.

**Fig. 4:**
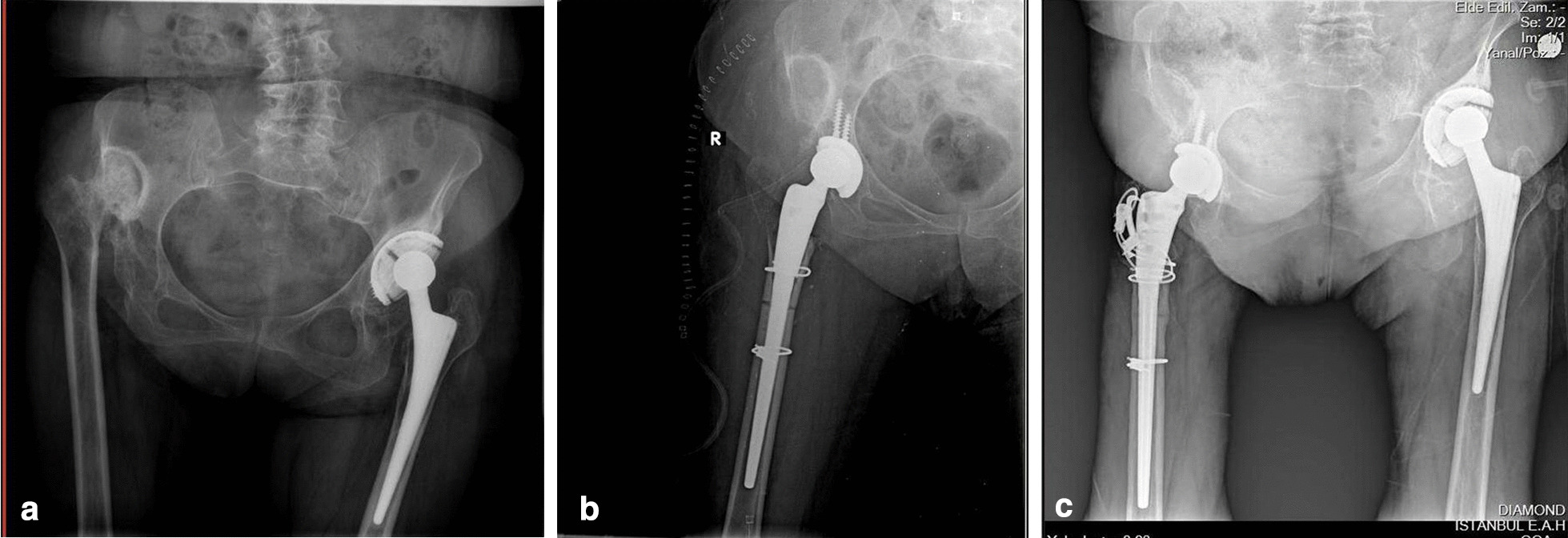
Radiographs of a 53 years old female with high hip dislocation. **a** Preoperative anteroposterior view. **b** Early postoperative radiographic image. Hip was reconstructed at the level of the anatomic hip center by cementless modular total hip arthroplasty combined with transverse osteotomy. Trochanter major fracture was observed and treated with trochanteric plate and cable grip, with bony union achieved at the 8th postoperative week (**c**)

Complications were observed in 9 (13.2%) hips, with revision surgery performed in 8 (11.7%) of these cases. Posterior hip dislocation was encountered in 1 patient, requiring open reduction and replacement of the femoral stem to adjust the anteversion angle. Femoral revision, using a larger femoral stem, was performed in 2 hips in the early postoperative period due to non-union and rotational instability at the osteotomy site. A two-stage revision was performed in 2 hips due to septic loosening. A revision was performed in 1 patient in the 3^rd^ postoperative year due to aseptic loosening of the femoral stem and in the 5^th^ postoperative year due to acetabular loosening. One case of sciatic nerve dysfunction was observed, although partial recovery was achieved. Fracture of the greater trochanter occurred in 1 hip, which was treated with a trochanteric plate and cable grip at the early postoperative period, with bony union achieved at the 8^th^ postoperative week (Fig. 4).

## Discussion

We provide evidence for the feasibility of using a cementless and modular THA, combined with sub-trochanteric transverse shortening osteotomy, as a treatment option for high hip Crowe type IV—Hartofilakidis type III dislocations. Here we show a low incidence of complications and good clinical outcomes. The strengths of our study include a mean follow-up of 12.9 years; analysis based on consecutive hips classified solely using Crowe type IV—Hartofilakidis type III; use of the same acetabular cup, modular and cementless femoral stem designs, and a uniform type of transverse osteotomy performed in all cases. Previous studies evaluating cementless THA with sub-trochanteric femoral osteotomy for high hip dislocations have included different types of osteotomy and implants. Only two, however, have exclusively evaluated outcomes for Crowe type IV—Hartofilakidis type III dislocations using a modular femoral stem [10, 23].

Placement of the acetabular component in the true acetabulum improves the lever arm of the hip abductor muscles and lowers the risk of loosening of the acetabular component, which is increased by a vertical displacement of the hip center [23–25]. We achieved implantation of the acetabular component in the true acetabulum in all cases, with only 1 case of acetabular loosening identified 5 years postoperatively. Reduction of the femoral head to the anatomical hip center performed without femoral shortening increases the risk for neural and neurovascular complications due to the magnitude of distraction required [3, 8, 12, 25, 26]. A leg lengthening of > 3 cm after implantation has been suggested as an indication for sub-trochanteric shortening osteotomy [10]. In our case series, the mean pull-down length of the proximal part and shortening length of the femur were 66.5 ± 4.5 mm and 35.5 ± 3.5 mm, respectively. Indeed, Kılıcoglu et al. [12] and Sofu et al. [27] have reported a pull-down length of 45 mm and 46 mm, respectively, without incidence of nerve palsy. We had 1 case of sciatic nerve dysfunction, where only partial recovery was achieved.

The non-union of the osteotomy is a common complication after sub-trochanteric shortening osteotomy due to poor resistance against applied rotational and axial bending force. Previously reported rates of non-union for high hip dislocations range between 2.8% and 7.1% [28–31]. The risk of a delayed union can be lowered by appropriate selection of the type of osteotomy and femoral stem implant [12]. Yasgur et al. [15] have suggested augmenting the osteotomy site with allograft struts and cable wires, regardless of the osteotomy type, to maintain rotational stability. Akiyama et al. [32] recommended additional plate fixation to maintain torsional stability with cementless conventional stems after transverse shortening osteotomies. In their evaluation of 44 patients with developmental dislocation of the hip treated by THA, using cementless non-modular stems with a transverse femoral shortening osteotomy, Yalçın et al. [33] used a plate and screws for additional stabilization of the osteotomy site in 10 cases. The 5 cases of non-union identified in their series occurred in patients in whom plate and screw stabilization had not been used. Based on their experience, these authors recommended performing internal fixation with plate and screws to provide additional rotational stability and lower the risk of non-union and malalignment at the osteotomy site [15, 17]. Of further concern is the relatively small contact surface with transverse osteotomies [23], with retraction of the gluteus medius muscle, and thus of the proximal femoral segment, enlarging the gap at the osteotomy site. Therefore, sufficient compression across the osteotomy site is necessary to prevent distraction [14]. In their biomechanical comparison of 4 types of sub-trochanteric osteotomies, Muratli et al. [34] could not identify specific features that could increase stability. Furthermore, in a meta-analysis by Li et al. [35], no significant differences between transverse and modified osteotomies with regard to the rate of complication and survival were found. The transverse osteotomy, however, provides a technically simpler method to correct the angle of femoral anteversion, with minimal periosteal damage at the osteotomy site [23, 35]. A transverse osteotomy also provides several other advantages, including preservation of the proximal femoral metaphysis and simultaneous shortening and correction of femoral anteversion [11]. In our case series, we exclusively used a transverse osteotomy, with a non-union rate at the osteotomy site of 2.9%. In our experience, the combination of transverse osteotomy and the use of a modular type of femoral stem provides the best option to lower the risk of non-union. Modular stems provide press-fit fixation for both the proximal and distal femoral components, individually, with the wide range of sizes of the proximal and distal components facilitating sufficient rotational stability and compression at the osteotomy site [36, 37]. By comparison, rigid fixation is not possible with conventional cementless femoral components due to the mismatch between the proximal and the distal cortices [23]. Moreover, the ability to place the proximal segment of the femoral component separately from the diaphyseal segment, including fixation of the proximal segment with a stepped sleeve with modular stem, is beneficial for correction of excessive anteversion [11], which is common even in cases of mild hip dysplasia and is associated with an increased risk for subluxation post-THA [1]. Additionally, in many of these cases, the proximal femur may be too narrow for a fit and fill style implant (broached body). This limitation is overcome with the use of modular femoral stems, which provide 3 different tapered stem sizes with 4 different vertical height options, allowing the proximal femoral canal to be prepared after the distal component is implanted to achieve the desired vertical offset and version correction. In our case series, we corrected femoral anteversion using a modular stem through in situ distraction and rotation of the proximal segment relative to the distal segment. In cases with a > 20° of anteversion, de-rotation was combined with a modular metaphyseal sleeve at the osteotomy site. With our approach, we encountered only 1 case of dislocation in the early postoperative period due to insufficient primary rotational stability. It has been suggested that modular femoral components have a risk of fretting, corrosion, and mechanical failure, resulting in osteolysis and loosening [10, 11]. In our case series, we identified only 1 case of osteolysis around the femoral stem with further aseptic loosening, with no evidence of gross metallic debris or blackening of the periprosthetic tissue observed during the stem revision surgery.

In this study, we used two types of modular stems. First-generation modular fluted tapered stems (Helios, Valencia, Biomet, Spain) were available in our hospital for use in 39 patients (47 hips) between 2001 and 2011. New generation cylindrical stems, anatomically bowed with a distal bullet-tip (Arcos, Zimmer, Biomet), were used in 17 patients (21 hips) after 2011. Broach proximal body was adapted to these stems. Cylindrical stems were superior to the fluted tapered stems to exhibit superior neutral alignment. These modular prostheses also had the versatility of high and standard offset. Therefore, hips achieving high offset did not have Trendelenburg gait after the operation.

### Future work

Ceramic inner cups and heads can also be used with the new generation modular prosthesis type. As the number of surgeries using the new generation modular stems increases, future work to compare the results of fluted tapered and cylindrical stems will be necessary. With a long-term study, the results of THA with a ceramic-ceramic articulation can also be evaluated.

There are limitations in our study that warrant discussion. First, this is a retrospective study with no control group, which limits the strength of our analysis. Moreover, we did not compare the results of THA combined with shortening osteotomy between non-modular and modular stems. Second, all THAs were performed by 3 senior surgeons. Third, the follow-up period is still mid-term, and the durability of these complex reconstructions must continue to be assessed in long-term follow-up studies.

## Conclusion

Cementless modular THA combined with sub-trochanteric transverse shortening osteotomy is a feasible option for treating high hip dislocations, with satisfactory and reliable 10-year clinical outcomes. Sufficient rotational stability of the cementless implant and union rates results in improved hip function, restoration of more normal limb lengths, and a low incidence of complications.

## Data Availability

The datasets used and/or analyzed during the current study are available from the corresponding author on reasonable request.

## Abbreviations

THA: Total hip arthroplasty
HHS: Harris hip score
